# Going Beyond Rate Changes as the Sole Indicator for Dynamic Triggering of Earthquakes

**DOI:** 10.1038/s41598-020-60988-2

**Published:** 2020-03-05

**Authors:** Kristine L. Pankow, Debi Kilb

**Affiliations:** 1 Department of Geology and Geophysics, University of Utah, Salt Lake City, UT USA; 2 Scripps Institution of Oceanography, Univ. of California San Diego, La Jolla, CA USA

**Keywords:** Natural hazards, Seismology

## Abstract

Remote earthquake triggering is a well-established phenomenon. Triggering is commonly identified from statistically significant increases in earthquake rate coincident with the passage of seismic energy. In establishing rate changes, short duration earthquake catalogs are commonly used, and triggered sequences are not typically analyzed within the context of background seismic activity. Using 500 mainshocks and four western USA 33-yearlong earthquake catalogs, we compare the ability of three different statistical methods to identify remote earthquake triggering. Counter to many prior studies, we find remote dynamic triggering is rare (conservatively, <2% of the time). For the mainshocks associated with remote rate increases, the spatial and temporal signatures of triggering differ. We find that a rate increase coincident in time with mainshock energy alone is insufficient to conclude that dynamic triggering occurred. To classify dynamically triggered sequences, we suggest moving away from strict statistical measurements and instead use a compatibility assessment that includes multiple factors, like spatial and temporal indicators.

## Introduction

Clear examples of remote dynamic triggering include increases in local earthquake rates over large areal extents as observed following the Mw 7.3 1992 Landers, California, earthquake^1^; the Mw 7.9 2002 Denali Fault, Alaska earthquake^2–5^; and the Mw7.4 1999 Izmit, Turkey, earthquake^6^. For these three examples, the rate increases were not subtle, but represented significant increases in rates of local earthquakes coincident in time with the passage of the surface waves from the triggering mainshocks. There are many other cases of documented remote dynamic triggering (see reviews and references therein^7–10^). In many of these examples, the seismicity rate change is subtle and the spatial extent very localized. Causality is assumed primarily because local events are either coincident with the passage of the surface waves, as determined by the presence of local earthquakes in the wavefield^11–15^ or the triggered events initiate within several hours after the passage of the mainshock seismic waves, leading to the concept of delayed dynamic triggering^16–19^. The prevalence of identified mainshocks generating remote, small magnitude earthquakes has led to two hypotheses: (1) remote dynamic triggering is ubiquitous^13^ and (2) dynamically triggered events are small, often below the magnitude of completeness (Mc) and to identify these events requires catalog enhancement^14,20–24^.

The difference between the obvious remote triggering following the Landers, Denali Fault, and Izmit earthquakes, and the subtler instances of triggering and instances of delayed dynamic triggering leads to the question: what are the indicators for dynamic triggering and do different indicators imply differences in the physical mechanisms generating the triggered earthquakes? Here, we step back and first ask whether statistically significant changes in rate are sufficient to be the sole indicator for dynamic triggering. Currently, within the statistics community there is a debate over removing “statistical significance” from the lexicon of scientific study^25^. This movement argues that we should abandon relying on a given significance value, like a p-value of 0.05 or 95% confidence interval (which were arbitrarily chosen to begin with), and instead move toward a compatibility assessment. In our analysis, we examine statistical significance in context of the spatial characteristics of the triggered events and the temporal behavior of the rate changes (factors also examined by Aiken *et al*.^21^).

In the initial cases of dynamic triggering, there was an increase in the rate of earthquakes over a large spatial extent^1–6^. For these examples, it appears there is something fundamental in the passing seismic energy, amplitude or frequency, and/or that large areas of the crust are critically stressed and primed for earthquakes^26,27^. This has led many to look for a triggering threshold^22,28,29^ or other characteristics of the dynamic wave^30,31^ that could be used to anticipate remote dynamic triggering. However, in many subsequent studies the change in earthquake rate is subtler and the spatial extent more localized. For these cases, it seems plausible that the driving force is spatially confined and perhaps related to the interaction between the incoming waves and the orientation of local faults^12,21,32–35^ or the activation of fluids^16,19,36^.

### Assessing dynamic triggering

One of the main challenges of remote dynamic triggering studies is how to determine the background seismicity rate and subsequently how to determine significant rate changes. Previously, researchers have used many different statistical approaches. Commonly, these statistics compare windows prior to and following the arrival of the transient mainshock energy. The windows range from as small as ± one to ± three hours^22,37^ to ± several days^4,38,39^. To identify rate changes, many statistical methods compare the number of events in the window prior to the mainshock energy to the number of events in the window post the mainshock arrival.

The most common statistic cited in dynamic earthquake triggering studies is the beta-statistic^40,41^. This statistic was originally developed to identify periods of earthquake quiescence. However, since the statistic measures differences in seismic rate between the number of earthquakes in a pre-window and the number predicted for a post-window normalized by the variance in the rate calculated using the pre-window, it can also be used to find periods of seismicity increases. Beta is defined as:

1 $$\beta =\frac{Npost\,-\,\Lambda }{\sqrt{\Lambda }}$$

where *T*_*a*_ and *T*_*b*_ are the window lengths for counting the number of earthquakes after (N_post_) and before (N_pre_) the time of interest, respectively, and $\Lambda$ = N_pre_ * $\frac{Ta}{Tb}$.

In originally deriving the beta-statistic, it was assumed that: (1) the dataset was stationary (declustered seismic catalog) and (2) the pre-event window used to determine the rate contain at least one earthquake. The second assumption sets limits on how small the windows can be, and Matthews and Reasenberg^40^ cautioned that significant anomalies found for short windows be treated “suspiciously.” They also suggested that when evaluating the significance of the beta-statistic, one look at the distribution of beta over an interval. Prejean and Hill^39^ investigated beta-statistic thresholds using Monte-Carlo resampling of data from Alaskan Volcanoes and found beta thresholds ranged from 2.7 to 16. Importantly, their minimum 2.7 value is larger than a beta threshold of 2, which is often used when assuming a Gaussian distribution^42^.

Alternative statistics to measure rate changes include the Z-statistic^43–45^. The Z-statistic, like the beta-statistic, examines the rate of events in two different time windows using a stationary catalog.

2 $$Z=\frac{Npost\ast Tb-Npre\ast Ta}{\sqrt{Npost\ast T{b}^{2}+Npre\ast T{a}^{2}}}$$

where *N*_pre_, *N*_post_, *T*_*b*_ and *T*_*a*_ are as defined above. Here, the difference in the mean number of events between the two-time windows is normalized by the sum of the variance, and the 95% and 99% significance thresholds are 1.96 and 2.57, respectively^44^. Note that when calculating the beta-statistic, if *T*_*b*_ and *T*_*a*_ are combined to predict the number of events in *T*a ($\Lambda$ = (N_pre_ + N_post_)* $\frac{Ta}{(Ta+Tb)}$) and the difference is normalized by the variance in this rate^39^, then the calculated value more closely approximates a Z-statistic^46^.

A third statistic assumes a Poisson distribution and calculates the difference from the mean (DFM)^13,23,24^. In this statistic, since a Poisson distribution assumes a constant rate, the number of events in the *T*_*b*_ window is taken to be the mean and the standard deviation is the square root of this value. For data sets with large number of samples generating the mean, 95% and 99% significance is equal to 1.96 and 2.58 times the standard deviation (σ), respectively, and significant rate changes are found when N_post_ > N_pre_ + [1.96 or 2.58] * (σ), for 95% and 99%, respectively.

The three statistics described above depend on the ability to reliably predict the rate of earthquakes based on a chosen time window. For comparison, we propose a fourth empirically derived statistic where we calculate the background rate over three-year windows using the distribution of the number of events in a shorter window (here 5-hours). However, we first construct a uniform catalog by correcting for Mc (see full explanation in the Methods section). Using the distribution of number of events in the 5-hour windows, we use percentiles to identify threshold rates that occur less than 5% and 1% of the time.

### Identified cases of dynamic triggering

**Figure 1:**
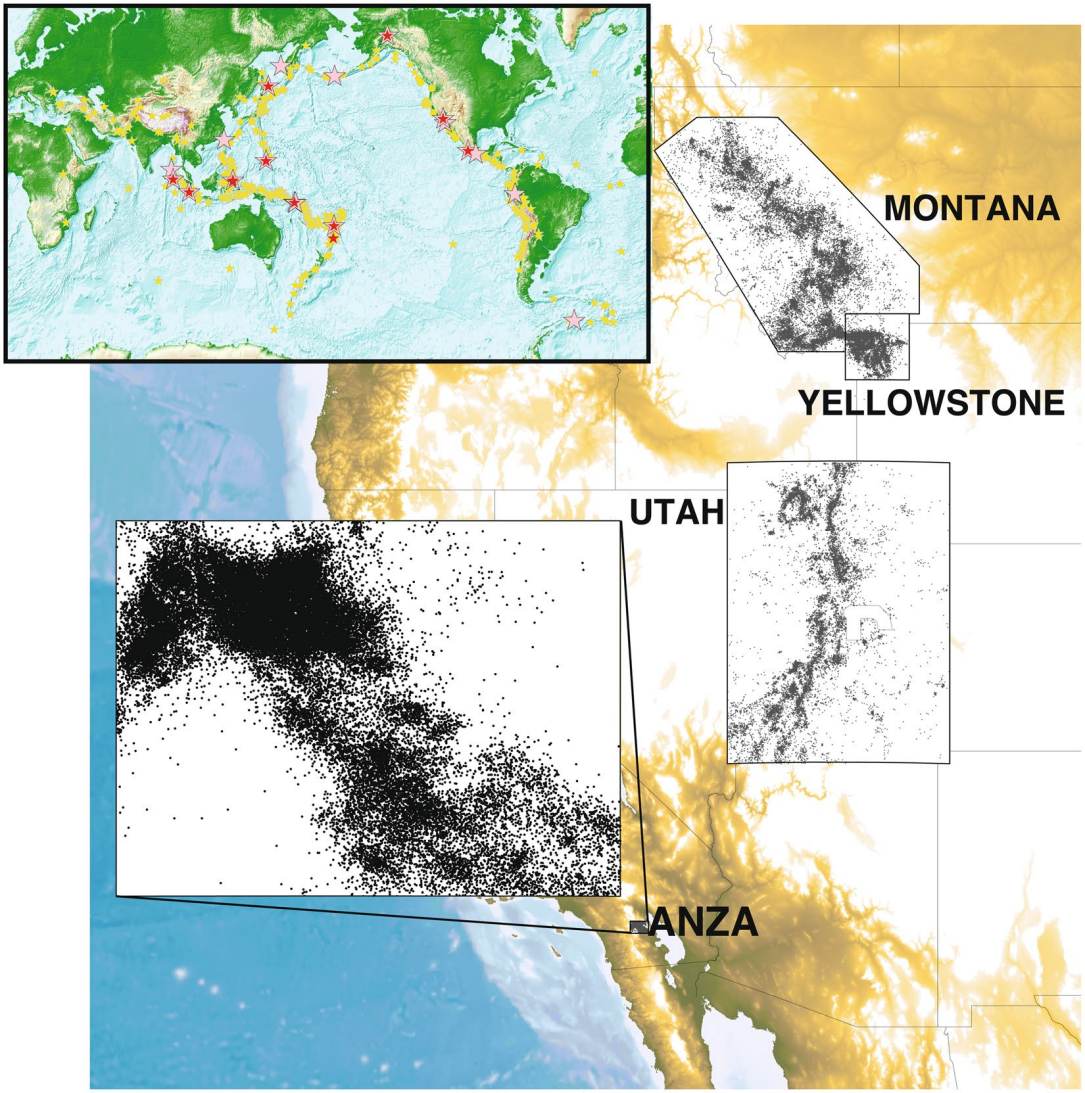
(top left inset) 500 mainshock earthquakes used in this study. Of these, 489 M ≥ 7.0 are teleseismic mainshocks (stars) and 11 are M ≥ 6.7 along the North American west coast (circles). Triggering mainshocks determined using the empirical method at the 95% significance level (pink, N = 19) and 99% (red, N = 12) are also shown. (basemap) Map of the four study regions (labeled). Figure generated using GMT-5.4.4^56^ (http://gmt.soest.hawaii.edu/doc/5.4.4/gmt.html).

**Figure 2:**
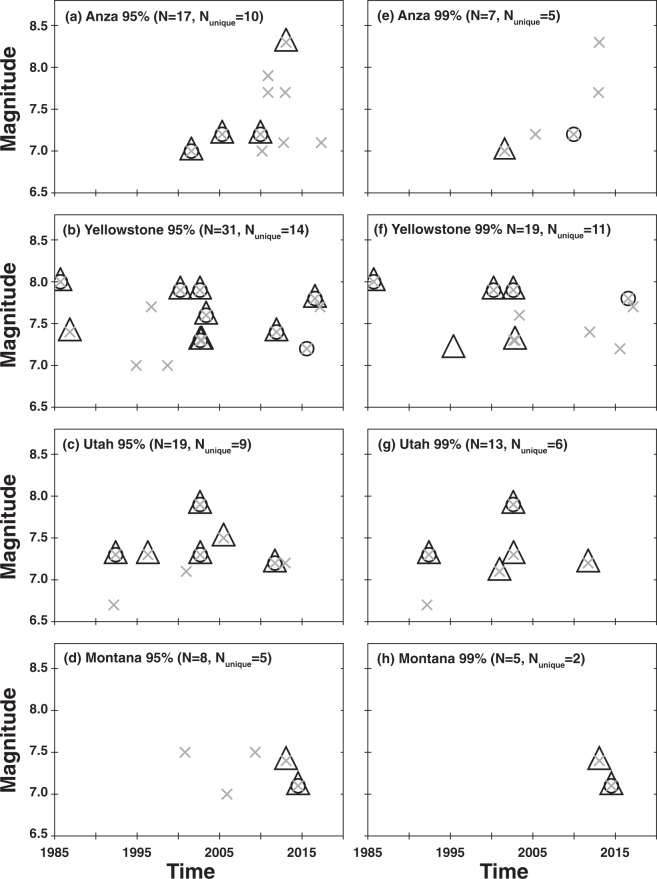
Statistical results. (**a–d**) 95% significant and (**e–h**) at 99% for methods: empirical (triangle), Z-statistic (circles), and difference from the mean (x). N is the total number of triggering mainshocks identified by any of the methods (different methods may identify the same mainshock as triggering). N_unique_ is the number of unique mainshocks.

Using 500 mainshocks (Supplementary Data 1) and 33-years of regional earthquake catalog data corrected for Mc from four regions (Fig. 1; detailed description of earthquake catalogs in Data and Methods section), we compare instances of significant rate changes identified using three statistical methods: DFM, Z-statistic, and the proposed empirical statistic. We do not include the beta-statistic, because given the short-duration (5-hours), for many (1570 of 2000; 78.5%) remote mainshocks N_pre_ is zero. Given this, it is not possible to correctly calculate the beta-statistic and we are careful to heed the warning regarding assigning significance when using small windows^40^. For the DFM method, if N_pre_ is zero, we assign a value of one to preclude the case when < 2 events can be called triggering. Using the three chosen statistics, we identify 38 and 24 rate increases coincident with a mainshock at the 95% and 99% significance levels, respectively (Fig. 2, Supplementary Table S1 and Supplementary Data Files 1 and 2).

Applying the DFM method using the short, 5-hour, pre-event time window identifies the most instances of rate increases coincident with the arrival of the seismic waves, 38 and 24, at the 95% and 99% confidence levels, respectively. However, because of the short 5-hour duration of the observation window (the duration used in many dynamic triggering studies^13,23,24,43^), this method is inherently plagued by the statistics of small numbers. In addition, there is no correction for variance in these data. The results from the proposed empirical method and the Z-statistic flag fewer triggering mainshocks than the DFM method.

If dynamic triggering preferentially triggers small magnitude quakes, as has been suggested in previous studies^20,34,47^, further enhancement of these regional catalogs may lead to more instances of potential dynamic triggering. However, the Mc levels for the regional earthquake catalogs used in this study are already relatively low (all time periods <2.5; majority of time periods <1.5; Supplementary Fig. S2). The different results between statistical methods can largely be attributed to the ability to more fully describe and characterize the seismicity rate within a 3-year catalog (empirical statistic) versus the rate within a 10-hour catalog (Z-statistic) or 5-hour catalog (DFM statistic). For example, for the Yellowstone and Anza catalogs, the long-term background rate can be high leading to high threshold values (>6; triggering less likely), while for the Utah and Montana catalogs the earthquake rate can be low leading to low threshold values (<4; triggering more likely).

Notably, all three statistical methods, identify previously documented cases of remote triggering in Utah and Yellowstone following the 2002 Mw 7.9 Denali Fault, Alaska, earthquake^2–5^, triggering in Utah following the 1992 Mw 7.3 Landers, California earthquake^1^, and in Anza following the 2010 Mw 7.2 El Mayor Cucapah earthquake^47^, (Fig. 2 and Supplementary Data Files 2 and 3). Newly identified cases of dynamic triggering include triggering in Yellowstone following the 1985 Mw 8.0 Mexico City earthquake and the 2000 Mw 7.9 Sumatra earthquake, and in Anza following the 2001 Mw 7.0 Guam earthquake and perhaps following the deep 2013 Mw 8.3 Sea of Okhotsk earthquake (Fig. 2). Other potential cases of remote triggering are discussed below.

### Triggering in a regional context

Thus far, we have identified increases in rates of local earthquakes coincident with seismic waves from distant mainshocks. Next, we look at these rate increases in the context of the cumulative time history of earthquakes over a plus/minus one-month time-period and also examine the spatial footprint of the triggered events. As previously noted, for the triggered earthquakes in Utah following the 2002 Denali Fault earthquake, what was unique was the widespread distribution of events over a short-time window (not just the rate increase) and that the widespread seismicity tended to form clusters^4^.

**Figure 3:**
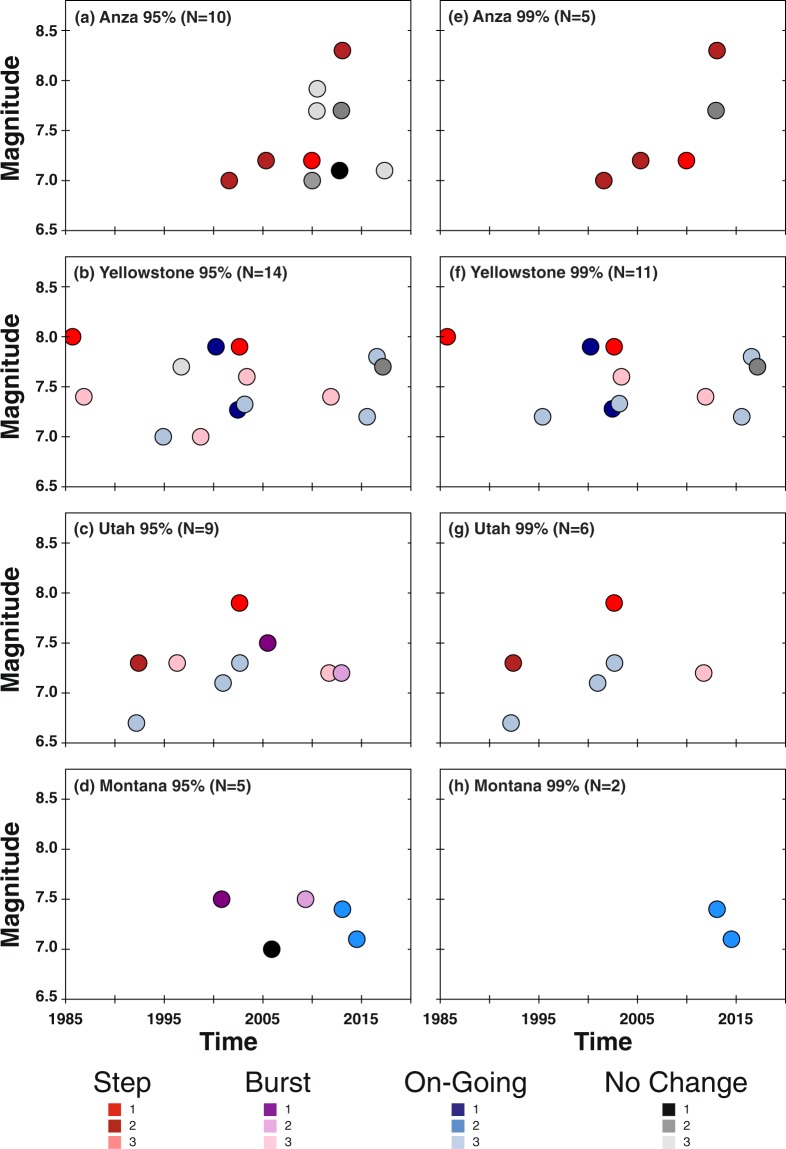
Temporal (color) and spatial (hue) behavior for mainshocks with a rate increase (Fig. 2). (**a–d**) 95% significance level and (**e–h**) at 99%. Temporal classification shown and labeled in the legend. Spatial patterns (numbers in legend): widespread (1, darker hue), single source zones (3, lightest hue) and somewhere between widespread and single source zones (2, middle hue). N is the number of triggering mainshocks shown in each panel.

**Figure 4:**
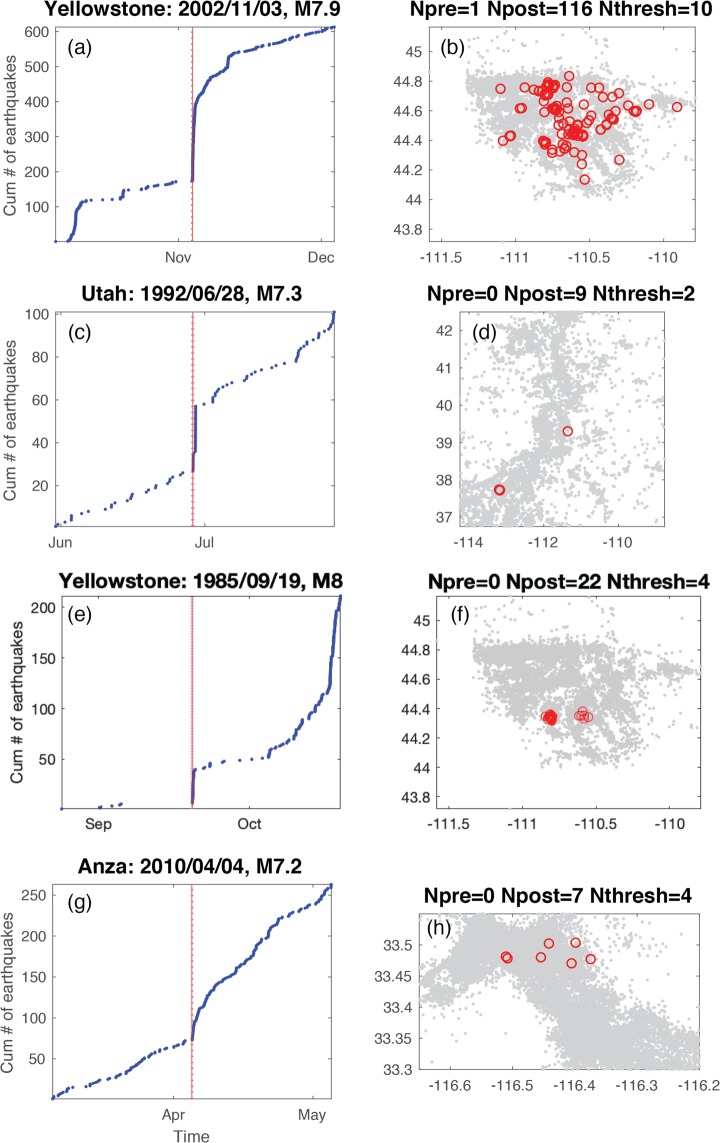
Remote triggering evidenced by temporal step-like increases and corresponding spatial distributions. (**a**,**b**) Yellowstone, spatial class 1. (**c**,**d**) Utah, spatial class 2. (**e**,**f**) Yellowstone spatial class 1. (**g**,**h**) Anza, spatial class 1.

**Figure 5:**
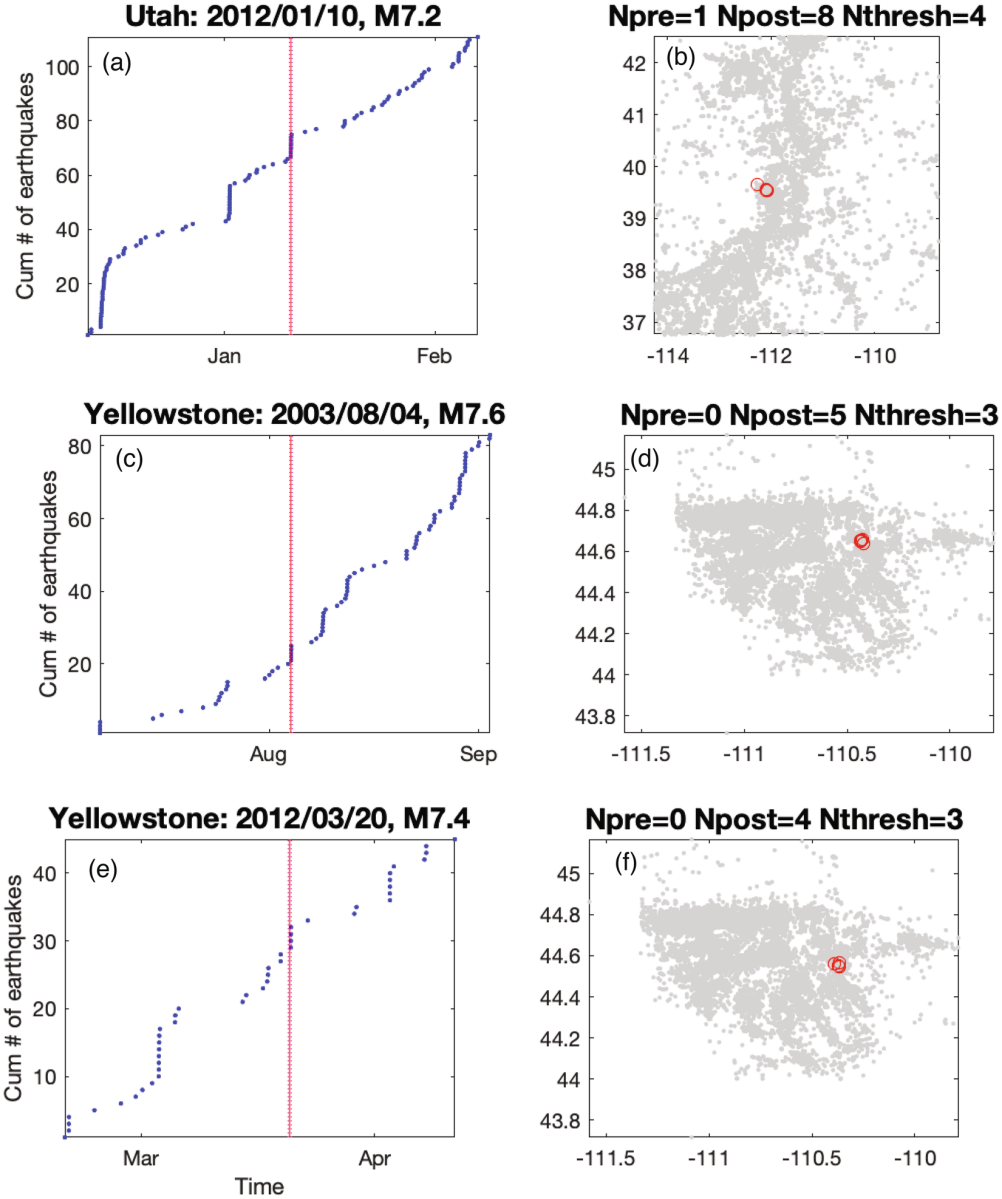
Remote triggering during temporal burst-like sequences and corresponding spatial distributions. (**a**,**b**) Utah, spatial class 3. (**c**,**d**) Yellowstone, spatial class 3. (**e**,**f**) Yellowstone, spatial class 3.

**Figure 6:**
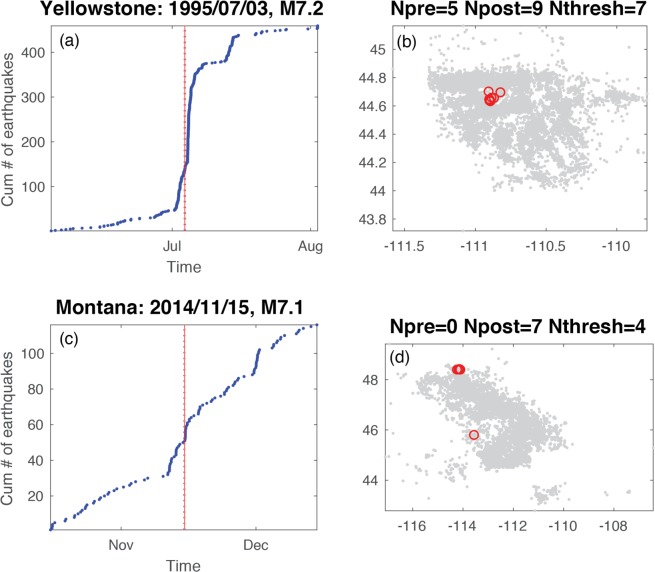
Remote triggering during on-going temporal sequences and corresponding spatial distributions. (**a**,**b**) Yellowstone, spatial class 3. (**c**,**d**) Montana, spatial class 2.

For each mainshock deemed capable of remote triggering, we categorize the time history into four classes (Fig. 3). The signature of class one is most consistent with triggering following the Landers and Denali Fault earthquakes, a rapid, step-like increase in seismicity compared to the background rate (Fig. 4). The signature of class two is where the variation in cumulative event time history over a two-month time window encompassing the mainshock shows multiple bursts of seismic activity (Fig. 5). In some cases, the burst cycle initiates with the dynamic energy of the remote mainshock, but in other cases cyclical bursts were already an ongoing feature of these data. The signature of class three is when there is a rate change during an ongoing sequence that is clearly visible in the cumulative time history (Fig. 6). For class four there is no visual change in the cumulative time history over the two-month period, which is often the case when small numbers represent the deemed increase (i.e., a rate change from 2 events in the pre-window to 4 events in the post-window). Only the DFM method flags such subtle rate changes. We also classify the spatial extent of the events occurring in the N_post_ window: widespread (Fig. 4b,f,h), single isolated clusters (Fig. 5b,d,f), a signature in between widespread and isolated (Fig. 6d), and too few to speculate (N_post_ < = 2). Of note, temporal class one sequences tend to exceed the statistical thresholds by the largest factors and the triggered events tend to have the largest spatial footprints. Temporal classes two and three sequences tend to activate single source zones.

When looking at the increases identified by multiple statistical measurements (Figs. 2 and 3), we find that Anza rate changes are often the result of step-increases (temporal class 1), while in Montana increases are primarily restricted to on-going sequences (temporal class 3). For Utah and Yellowstone, there is a mix of temporal types and variability in the spatial extent of the rate increase events. With three exceptions, bursty sequences are only significant at the 95% level, and it is not clear whether these small bursts of seismicity are the result of dynamic energy or the results of ongoing background seismicity. One of the exceptions is in Utah following the 2012 Mw 7.2 Sumatra quake (Fig. 5a,b), when there were three distinct local sequences during the two-month time period, the first located in southern Utah, the second in northern Utah, and the third in central Utah that was coincident with the mainshock’s seismic wave arrival in the region. Given that the bursty nature of the cumulative time plot corresponds with spatially distinct regions, this 2012 rate increase could be argued to be a step increase or just background seismicity. The other exceptions are in Yellowstone following the 2003 Mw 7.6 Scotia Sea quake (Fig. 5c,d) and the 2012 Mw 7.4 Oaxaca, Mexico quake (Fig. 5e,f). For the 2003 mainshock, there were 5–6 sequences within the two-month time frame. Spatially, there are local clusters of events, but the seismicity is diffuse for this two-month period. The cluster corresponding to the rate increase began ~1 hr after the origin of the mainshock with an M_L_ 2.7 quake followed by four smaller shocks. For the 2012 event, there were 3 larger and several smaller sequences within the two-month time period. Again, the seismicity is quite diffuse (as is typical in Yellowstone) with a few localized clusters. One of these clusters began ~3 hr after the origin of the mainshock with an M_L_ 2.0 quake followed by three smaller shocks. These examples demonstrate the required additional scrutiny needed to truly assess if triggering occurred.

After close examination, we find that only 24 to 38 of the 2000 (500 in four regions) mainshocks or 1%-2% are coincident with significant increases in earthquake rates at the 99% and 95% levels, respectively, and may represent remote dynamic triggering. Of these, only 8 are consistent with a temporal step change coincident with the arrival of the mainshock energy. Notably, the rate change for these eight events is significant at both the 95 and 99% levels. Eleven and three (95% and 99% levels, respectively) of the sequences are bursty and as discussed not easily associated with dynamic triggering. For the remaining 11 sequences, the rate increase is within an ongoing sequence. While difficult to conclusively associate with the passage of dynamic stresses, it is notable that like the step increase case all 11 events are significant at both the 95 and 99% levels. Overall, we find that Yellowstone and Utah are more susceptible to remote triggering than Anza and Montana. Yellowstone and Utah exhibit all three temporal classes, while rate changes in Anza exhibit step increases and rate changes in Montana exhibit enhancements within on-going sequences. Limiting our mainshocks to only shallow events (≤100 km)^14,29,44^ that can generate larger surface waves does not appreciably change these results (see Supplementary Table S2).

### Implications for dynamic triggering

There is precedent in the seismological community that analysis using only changes in local earthquake rates can be used as an indicator for remote dynamic triggering. Here, we explore whether an increase in earthquake rate is always indicative of dynamic triggering. For example, should temporally bursty sequences be treated the same as temporal step increases? Can deep earthquakes trigger remote crustal earthquakes, as suggested by the Anza rate increase following the ~600 km deep 2013 Mw 8.3 Sea of Okhotsk earthquake? And, should increases in on-going sequences constitute remote dynamic triggering?

A second issue is the appropriateness of the statistics and the window length used to determine the earthquake background rate. Many of the statistics, beta-, Z-, and DFM, assume that the catalog represents independent points (stationary). However, declustering may be counterproductive because the goal of dynamic triggering studies is to identify changes in what could be termed remote aftershocks, so these statistics should be used with caution, especially when using short window lengths. We propose using an empirical approach to describe the rates. A potential disadvantage to the empirical approach is the need for a longer duration catalog. However, because a longer catalog is used the distribution of earthquake rates can be established using a statistically significant pool—thousands of windows each with a rate measurement. The longer catalog can also be used to establish Mc. Not correcting for Mc can significantly change the triggering results^48^. When applying the Z-statistic and DFM to catalogs not corrected for Mc, we find 30–50% additional events (false positives) are flagged using the Z-statistic and 100–200%, using DFM (see Supplementary Table S1). These findings suggest it is essential to apply these statistics to catalogs that contain only events above the Mc level.

A third issue raised here is what constitutes a significant increase. If significance is set at 95% more sequences are identified as potentially triggered. However, many are bursty or within on-going sequences. Whereas if significance is set at 99% some triggered sequences may be missed, including the recently established triggering in Anza following the 2010 El Mayor Cucapah earthquake^47^. We suggest following the lead of the statisticians who have recently called into question the usefulness and validity of statistical significance. In doing so, we contend that the seismological community should also transition from using hard-set rules of statistical significance and instead transition to the concept of compatibility^25^. However, if following this path, then we will also need to define indicators of dynamic triggering beyond rate. These could include the spatial and temporal classes identified in this and other papers^21^. Our findings suggest down-weighting the link to dynamic triggering for bursty sequences and increasing the weight for widespread spatial triggering. Other factors to consider include peak ground velocity and strain changes^14,17,29,30,35,47–50^ and the orientation of incoming seismic waves with respect to the local faults^12,21,32–35^.

Moving forward, future research on remote earthquake triggering should de-emphasize use of the beta-statistic and DFM and move beyond exploring only narrow time window rate changes. Additionally, a change in rate should be one of many indicators used to identify remote earthquake triggering. Here, we place the most confidence in sequences exhibiting either a strong step increase in rate and/or that the triggered seismicity has a distinct spatial footprint.

## Data and Methods

### Data

The mainshock earthquake catalog includes global and regional seismic data from 01 January 1985 through 31 December 2017 (33 years). These data include 11 earthquakes M ≥ 6.7 from within the western US and Canada (24 ≤ Latitude ≤ 51; −131 ≤ Longitude ≤ −109) and 489 global earthquakes M ≥ 7 (Fig. 1). We search for remote triggering in four locations: Anza, CA, Montana, Utah, and Yellowstone (Supplementary Table S3). For the Utah catalog, we exclude mining induced seismicity (MIS, see Supplementary Table S4 for polygon boundaries) in central Utah. MIS is often strongly correlated with production rate^51^, and to fully understand triggering in the MIS region, one would first need a means to account for variations in rate that are the result of production efforts. In the four regional catalogs, the reported magnitudes are typically local (M_L_) or duration (M_d_). In rare instances, for some of the larger earthquakes, moment (M_w_) or body (M_b_) magnitudes are also reported. For less than 1% of the data the regional catalogs contain events that do not have assigned magnitudes, for these events we assume the magnitudes are below the Mc.

### Magnitude of completeness

We divide the 33-year regional catalogs into 3-year sub-catalogs, netting a total of 44 sub-catalogs for the four study regions. For each 3-year sub-catalog, we create histograms of the earthquake magnitudes and determine Mc for each sub-catalog from the mode of the histogram (Supplementary Fig. S1). When computing Mc, we use only the M_L_ and M_d_ events for each of the four target catalogs. We are careful, however, to check if the catalog for a given region has different M_L_ and M_d_ magnitude distributions. We take this cautionary step because previous work found that in some catalogs, especially for small magnitude events (<1.5), M_d_ is consistently larger than M_L_^52,53^. If this is the case in our data, then when the two different distributions are combined they would produce a double peak in the magnitude histogram. To help negate these limitations, when there are multiple peaks, we choose the larger value as the completeness level, recognizing that this approach will underestimate the completeness level. This method of calculating Mc approximates the Maximum Curvature Technique^54^.

We determine Mc for our 3-year catalogs to appropriately account for changing Mc levels (Supplementary Fig. S2). We evaluate data in 3-year blocks, and discard all data below the Mc level. For the other statistical methods, we follow two approaches: (1) use the Mc corrected catalogs in order to directly compare the empirical statistic with the other statistics and (2) use all available data to mimic how the Z-statistic and DFM are typically applied.

### Proposed empirical statistical method

In the new empirical method, the first goal is to establish the expected number of events within a chosen window length, here we use 5-hours to overlap with the arrival of mainshock energy and to be consistent with previous studies^13,23,24,48^. Using the Mc refined three-year catalogs, we count the number of events in each 5-hour time window incorporating a 1-hour sliding window. In this way, the first window spans 00:00 to 05:00, the second 01:00 to 06:00 and the third 02:00 to 07:00 and so on. The 3-year time span will increase when the dataset contains a leap-year, but in all cases the number of windows exceeds 26,000. We assign the number of events within each window (N_count_) to a timestamp at the start of the window (Supplemental Fig. S3).

We next build a histogram of N_count_ values. For all regions and all 3-year sub-catalogs, 5-hour windows predominantly have zero earthquakes (Supplemental Fig. S4). The distributions are right skewed making traditional measurements of mean and standard deviation less meaningful. To identify outliers, we use the empirical distribution of N_count_ values to compute a cumulative sum of the percentages for increasing N_count_, and identify threshold values (N_thresh_) when the cumulative sums equals or exceeds 95% and 99% (Supplemental Fig. S4). With the acquired information, we catalog timestamps that have elevated seismicity rates with respect to the expected rate over the associated 3-year window. Using this method we avoid having to decluster the catalog, because we are assuming that the 3-year time-window is long enough such that elevated earthquake rates, like aftershock sequences and swarms, will be averaged out.

To determine time periods coincident with the arrival of the mainshocks, we establish when the first teleseismic waves arrive at the centroid of each study region using the travel time toolkit TauP^55^. Using this as the onset, we next determine when the N_count_ rates within ± 5 hours of the arrival of the seismic waves from the remote mainshocks contain elevated rates at the 95% and 99% levels (Supplemental Fig. S3). All mainshocks associated with elevated counts are flagged for further analysis. For flagged cases, we compare the number of events in the 5-hour pre-window (N_pre_) to the number of events in the 5-hour post-window (N_post_). If N_pre_ > N_post_, we rule out triggering. Importantly, for triggering cases not only do we require N_thresh_ < N_post_, but we also require that N_pre_ < N_thresh_. Given this secondary restriction, our 95% level results will not necessarily be a subset of the 99% level results.

## Supplementary information

Supplementary Information.

Supplementary Data 1.

Supplementary Data 2.

Supplementary Data 3.

## Data Availability

All data used in this work came from catalog holdings from the ANSS (last accessed June 2018) and USGS COMCAT (last accessed October 2018). Data for the mainshocks and for Anza, CA, Yellowstone, and Utah regional catalogs were retrieved from the COMCAT catalog (last accessed October 2018) https://earthquake.usgs.gov/earthquakes/search/. The Montana regional catalog was retrieved from the ANSS catalog search: http://www.quake.geo.berkeley.edu/anss/catalog-search.html.
