# Supplementary material for: Going Beyond Rate Changes as the Sole Indicator for Dynamic Triggering of Earthquakes
Source: Sci Rep. 2020 Mar 5;10:4120. doi: 10.1038/s41598-020-60988-2 (PMC7058025; doi:10.1038/s41598-020-60988-2)
Supplement: Supplementary file 1 — Supplementary Information. [file 41598_2020_60988_MOESM1_ESM.docx]

**Supplementary Information for “Going Beyond Rate Changes as the Sole Indicator for Dynamic Triggering of Earthquakes”**

Kristine L. Pankow^1^ and Deborah Kilb^2^

^1^ Dept. of Geology and Geophysics, University of Utah, Salt Lake City, UT, USA

^2^ Scripps Institution of Oceanography, Univ. of California San Diego, La Jolla, CA, USA

**Description of Additional Supplementary Files**

File Name: Supplementary Data 1

Description: List of the 500 mainshocks used in this study, provided in csv format that includes a 1-line heading of the parameters.

File Name: Supplementary Data 2

Description: Data file in csv format of the results from the 95% level test. These data only include the 38 events that were identified as being triggered by one of our three methods (empirical, Z-statistic and difference from the mean (DFM)). Note that these 95% results are not necessarily a subset of the 99% results because for the empirical method, we enforce the rule that N_pre_ < N_thresh_ and N_thresh_ < N_post_.

File Name: Supplementary Data 3

Description: As in Supplementary Data 2, but for results from the 99% level test. These data only include the 24 events that were identified as being triggered by one of our three methods (empirical, Z-statistic and difference from the mean (DFM)).

**Table S1.** Number of triggered mainshocks for the three different methods (empirical, Z-statistic and difference-from-the-mean (DFM)). Asterisks indicate the results were derived using a catalog restricted to events at or above the magnitude of completeness.

| **Region** | **Threshold** | **Empirical*** | **Z*** | **Z** | **DFM*** | **DFM** |
| --- | --- | --- | --- | --- | --- | --- |
| Anza | 99% | 1 | 1 | 2 | 5 | 11 |
| Montana | 99% | 2 | 1 | 1 | 2 | 9 |
| Utah | 99% | 5 | 2 | 3 | 6 | 13 |
| Yellowstone | 99% | 5 | 4 | 4 | 10 | 15 |
| Total  (all regions) | 99% | 13 | 8 | 10 | 23 | 48 |
| **Unique** | **99%** | **12** | **7** | **9** | **21** | **47** |
| Anza | 95% | 4 | 3 | 4 | 10 | 34 |
| Montana | 95% | 2 | 1 | 2 | 5 | 28 |
| Utah | 95% | 6 | 4 | 6 | 9 | 32 |
| Yellowstone | 95% | 9 | 8 | 10 | 14 | 37 |
| Total  (all regions) | 95% | 21 | 16 | 22 | 38 | 131 |
| **Unique** | **95%** | **19** | **14** | **21** | **36** | **120** |

**Table S2.** Empirical method’s dependency on mainshock depths. Comparison of results derived from all mainshock depths (N=500; max depth 675 km) and only shallow (N=391, restricted to depths ≤100 km) mainshock events. Of the 500 mainshock events, 391 (78.2%) are shallow (depths≤100km). All results are computed using a ±5-hour time window with respect to the mainshock arrivals.

| Percentage | Regions | Depth Restriction | Total number of events | Number of triggered events | Triggered  (%) |
| --- | --- | --- | --- | --- | --- |
| 99 | All 4 regions | None | 2000 | 13 | 0.65% |
| 99 | All 4 regions | ≤100km | 1564 | 12 | 0.77% |
| 95 | All 4 regions | None | 2000 | 21 | 1% |
| 95 | All 4 regions | ≤100km | 1564 | 16 | 1% |

**Table S3.** The spatial extent of the four regional earthquake catalogs (Anza, Montana, Utah and Yellowstone). Information includes the spatial constraints, the number of events in each catalog (only M_c_ and M_L_ data), the catalog source and when it was last accessed.

| Catalog | Polygon Parameters | # of Events | Catalog Source |
| --- | --- | --- | --- |
| Anza | Lat Range:  33.3 33.55  Long Range:  -116.65 -116.2 | 42,543 | USGS ComCat  (October, 2018) |
| Montana | Lat. Long.  45.1667 -110.0000  45.1667 -111.3333  44.5000 -111.3333  44.5000 -113.0000  47.9500 -116.0500  48.5000 -115.0000  48.5000 -113.0000  46.0000 -109.5000  45.1667 -109.5000  45.1667 -110.0000 | 36,425 | ANSS Composite Catalog  Network Code: MB  (June, 2018) |
| Utah | Lat. Long.  36.7500 -108.7500 36.7500 -114.2500 42.5000 -114.2500 42.5000 -108.7500 36.7500 -108.7500 | 24,842 | USGS ComCat  Network Code: UU  (October, 2018) |
| Yellowstone | Lat. Long.  44.0000 -109.7500 44.0000 -111.3333 45.1667 -111.3333 45.1667 -109.7500 44.0000 -109.7500 | 41,480 | USGS ComCat  Network Code: WY  (October, 2018) |

**Table S4**. Polygon descriptors that outline regions in Utah that have mining induced seismicity for two different regions: Sufco and Crescent, Utah. All seismicity within these polygons were removed from consideration.

| Crescent | -111.300000000000000 39.166699999099997  -111.300000000000000 39.211549635899999  -111.300000000000000 39.256295398600002  -111.300000000000000 39.307433413100000  -111.300000000000000 39.358571427599998  -111.300000000000000 39.411307505099998  -111.300000000000000 39.451259079000003  -111.300000000000000 39.500799030600000  -111.300000000000000 39.542348667399999  -111.300000000000000 39.583299999099999  -111.338170635000000 39.611913668400000  -111.366699999999990 39.633299999099997  -111.366699999999990 39.679782081399999  -111.366699999999990 39.719733655200002  -111.366699999999990 39.749999999099998  -111.328920426000000 39.773590992499997  -111.287539781000010 39.799430630499998  -111.261964316000000 39.815400917500000  -111.233300000000000 39.833299999099999  -111.161421550000000 39.833299999099999  -111.086312591000000 39.833299999099999  -110.990428814000000 39.833299999099999  -110.881760533000000 39.833299999099999  -110.800259322000000 39.833299999099999  -110.701179419000000 39.833299999099999  -110.598903390000000 39.833299999099999  -110.500000000000000 39.833299999099999  -110.426962291000000 39.778528563599998  -110.374032080000010 39.738835866899997  -110.311640772000000 39.692048234700003  -110.233300000000000 39.633299999099997  -110.213772792000000 39.555132527900000  -110.201466952999990 39.505872216000000  -110.186436881000010 39.445706794600000  -110.166700000000010 39.366699999100000  -110.258515981000000 39.366699999100000  -110.343213316999990 39.366699999100000  -110.437499031000000 39.366699999100000  -110.516700000000000 39.366699999100000  -110.528724776000000 39.420865656200000  -110.540820882000010 39.475352620899997  -110.550000000000000 39.516699999099998  -110.587961092000000 39.541982086399997  -110.618579388000000 39.562373871699997  -110.650000000000010 39.583299999099999  -110.723552299999990 39.583299999099999  -110.789072881000000 39.583299999099999  -110.870574092000000 39.583299999099999  -110.950000000000000 39.583299999099999  -110.950000000000000 39.497602904600001  -110.950000000000000 39.419297819900002  -110.950000000000000 39.371355931300002  -110.950000000000000 39.317021790799998  -110.950000000000000 39.224334139500002  -110.950000000000000 39.166699999099997  -111.043164891000000 39.166699999099997  -111.121469976000000 39.166699999099997  -111.209363438000000 39.166699999099997  -111.300000000000000 39.166699999099997 |
| --- | --- |
| Sufco | -111.483300000000000 39.033299999100002  -111.455496161000000 39.033299999100002  -111.427304162000000 39.033299999100002  -111.403945077000000 39.033299999100002  -111.377766792000000 39.033299999100002  -111.355213193000000 39.033299999100002  -111.326215708000010 39.033299999100002  -111.298426452000000 39.033299999100002  -111.266700000000000 39.033299999100002  -111.266700000000000 39.001886007499998  -111.266700000000000 38.979735151000000  -111.266700000000000 38.960806237299998  -111.266700000000000 38.931808752499997  -111.266700000000000 38.903299999100000  -111.320577308000000 38.903299999100000  -111.349172050000010 38.903299999100000  -111.379377764000000 38.903299999100000  -111.412805420000000 38.903299999100000  -111.435761762000000 38.903299999100000  -111.483300000000000 38.903299999100000  -111.483300000000000 38.918115495800002  -111.483300000000000 38.936238923799998  -111.483300000000000 38.967250122899998  -111.483300000000000 39.001483264699999  -111.483300000000000 39.033299999100002 |


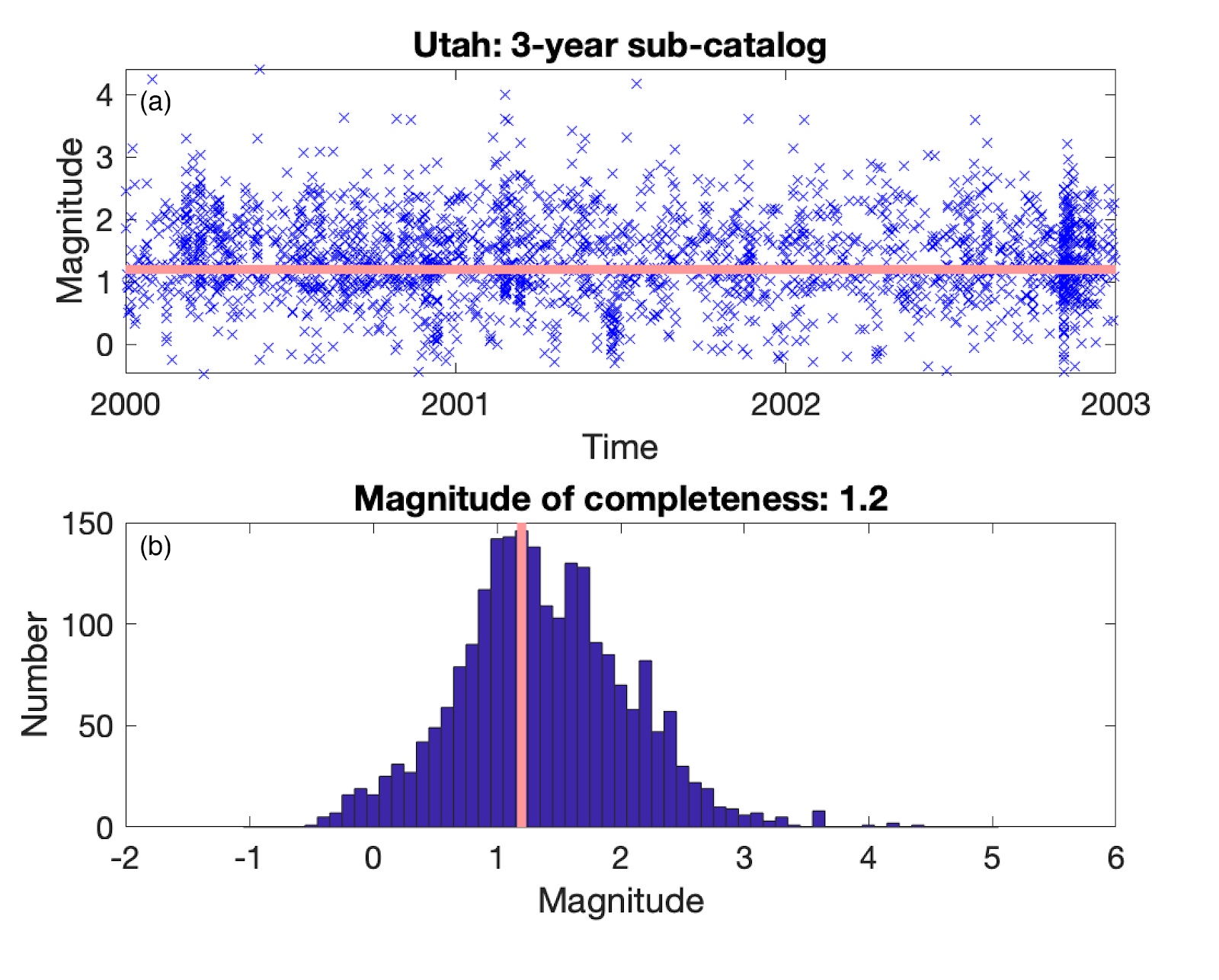


**Figure S1**. Establishing magnitude of completeness. (a) Example from a Utah 3-year sub-catalog (2000 through 2002) showing magnitude as a function of time. (b) Histogram of the events shown in (a). The magnitude of completeness level is assigned the mode of the histogram, in this example a value of 1.2.


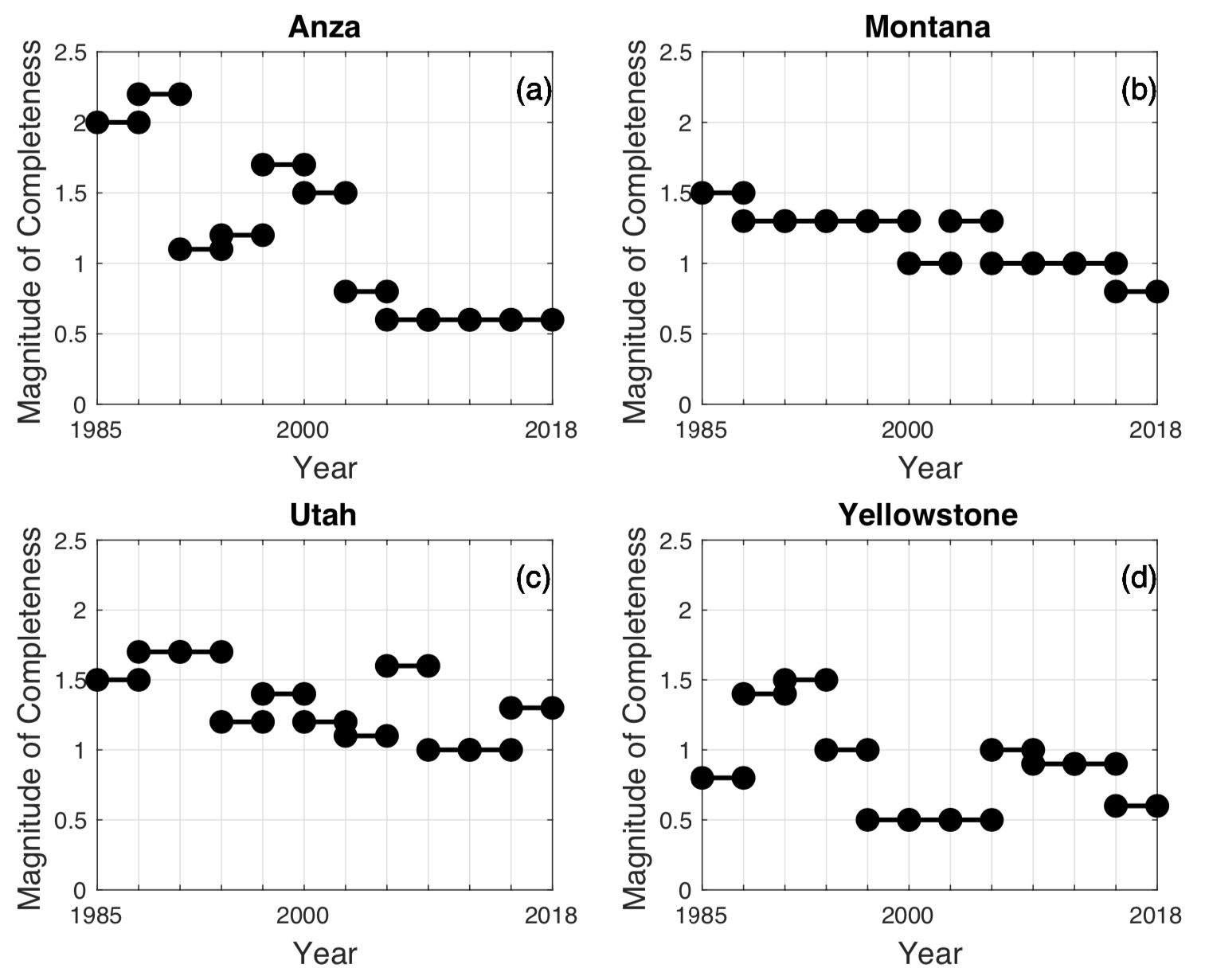
**Figure S2.** Magnitude of completeness for each catalog as a function of time for (a) Anza, (b) Montana, (c) Utah and (d) Yellowstone.


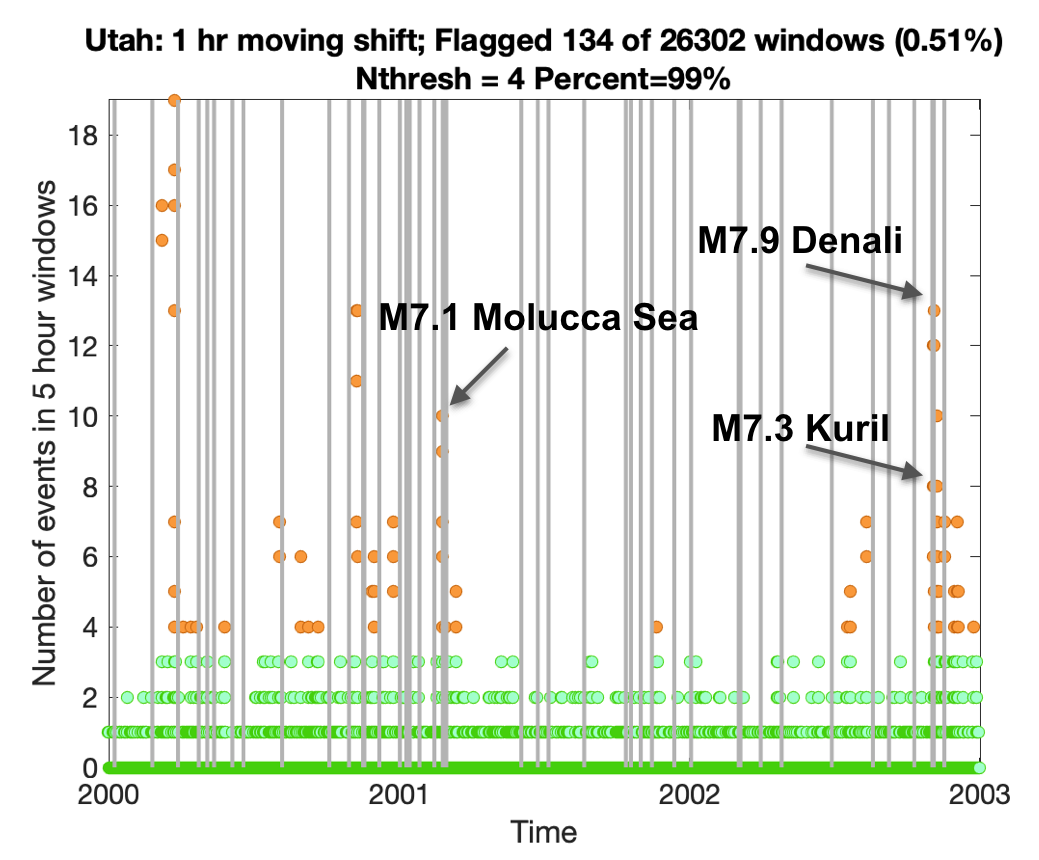


**Figure S3.** Sample results from the 2000 through 2002 Utah catalog. Each green dot represents a measurement of the number of earthquakes within a 5-hour window, assigned to the start of the window. Using a 1-hour shift between measurements, this nets >26,000 datum. The orange dots indicate times when the seismicity rate is elevated at the 99% level. Vertical lines represent the times when seismic waves from a large remote earthquake traverse the region. We have highlighted three instances when the seismic energy from large earthquakes are coincident with the start of an elevated rate of seismicity.


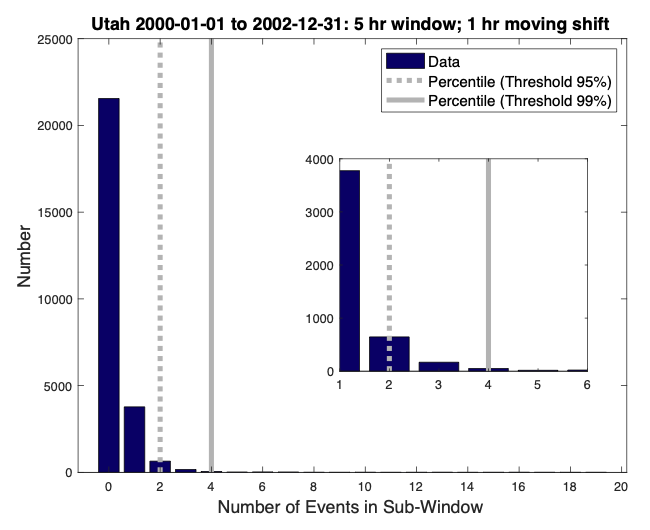


**Figure S4.** Example of the skewed nature of the number of events in a given 5-hour time window using the 3-year Utah catalog from 2000 through 2002. Data include >26,000 values representing the number of events in a 5-hour window over the duration of the catalog collected using a 1-hour sliding window. Using percentile measurements, we find the 95% level is 2 and the 99% level is 4 (more clearly shown in the inset figure, which zooms into these smaller values of the histogram). These percentile levels are assigned as triggering thresholds (N_thresh_=2 at the 95% level; N_thresh_=4 at the 99% level). These N_thresh_ values are specific to this 3-year Utah catalog and are not applicable to data from other time periods or other regions.
